# Solitary Fibrous Tumor of the Kidney: A Case Report

**DOI:** 10.1155/2013/147496

**Published:** 2013-04-10

**Authors:** Abdullah Demirtaş, Volkan Sabur, Hülya Akgün, Emre Can Akınsal, Deniz Demirci

**Affiliations:** ^1^Department of Urology, Erciyes University Medical Faculty, 38039 Kayseri, Turkey; ^2^Department of Pathology, Erciyes University Medical Faculty, 38039 Kayseri, Turkey

## Abstract

Solitary fibrous tumor is a spindle cell neoplasm mostly originating from pleura; however, it has also recently been reported to be extrapleural. A 57-year-old man presented with left lumbal pain. Ultrasonography and computed tomography showed a cystic lesion of 14 × 11 cm with solid areas and septations in middle and lower poles of the left kidney. Radical nephrectomy was performed. Immunohistochemical studies showed strong reactions with CD34 and CD99. A nuclear positivity with Ki-67 was observed in less than 1% of cells. Despite repeated stainings with vimentin, no clear tumor evaluation could be made due to artifacts. The tumor was negative with Bcl-2, desmin, HMB-45, S100, FVIII, and CD31. Histopathological and molecular studies made the diagnosis of a solitary fibrous tumor. The patient is now currently free of disease at the 26th month of followup.

## 1. Introduction

Although fibrous tumors mostly originate from pleura, tumors originating from urogenital system organs such as kidney, prostate, and urinary bladder have also been reported [1]. Solitary fibrous tumor (SFT) of the kidney is a rare mesenchymal cell tumor. SFT of the kidney was first defined by Gelb et al. in 1996 [2]. Nearly 50 cases of SFT of the kidney have been reported so far in the literature. Most of these types of tumors have a benign character [3]. It is difficult to differentiate it from renal cell carcinoma with imaging techniques. Definitive diagnosis can be made by pathological examinations including immunohistochemical and molecular techniques. In this paper, we present a case who was operated with radical nephrectomy and whose pathology result was reported as SFT. 

## 2. Case Report

**Figure 1:**
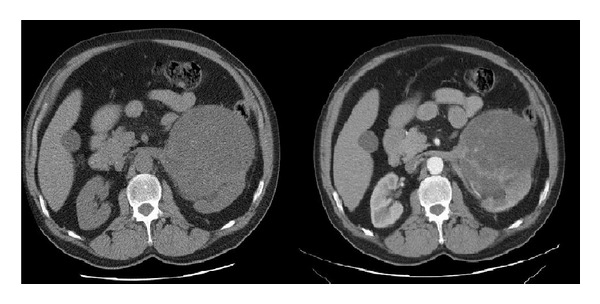
The view of the cystic, thick-walled, septated, and vascularized mass at the arterial phase of the computed tomography.

**Figure 2:**
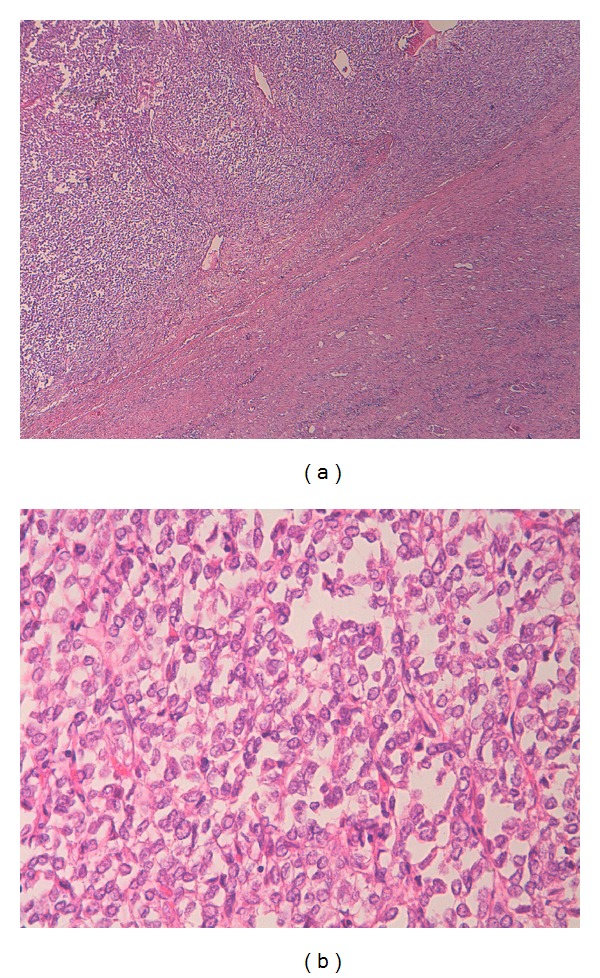
(a) Tumor tissue and intact parenchymal tissue (×40 hematoxylin-eosin). (b) The tumor cells with ovoid, round nuclei having coarse chromatin structure and a narrow eosinophilic cytoplasm (×400 hematoxylin-eosin).

**Figure 3:**
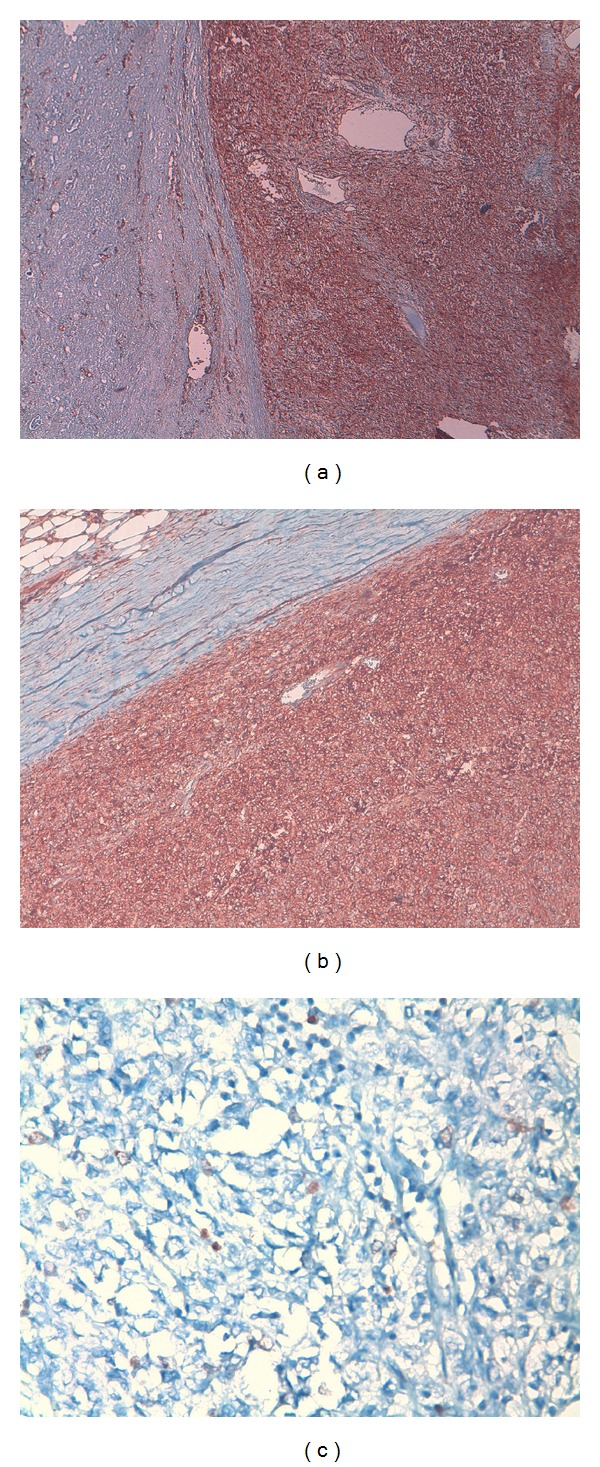
Immunohistochemical staining: (a) CD34 positive (×40), (b) CD99 positive (×100), and (c) staining showing a proliferative index of 1% with Ki-67 (×400).

A 57-year-old male patient presented with a left lumbal pain for 3 days. Physical examination with palpation revealed firm and mobile mass in upper left quadrant of the abdomen. No costovertebral tenderness was present. Urinalysis showed 3 erythrocytes. Laboratory data were as follows: blood urea nitrogen 16 mg/dL, creatinine 0.9 mg/dL, and hemoglobin 13.3 g/dL. Ultrasonography demonstrated a 14 cm vascularized, cystic, space-occupying formation with septations and solid components in middle and lower poles of the left kidney. Computed tomography identified a 14 × 11 cm cystic, space-occupying formation with many thick septae, which originated from anterior cortex of the left kidney and reached the level of pelvic inlet (Figure 1). Doppler ultrasonography showed patent main renal vessels and vena cava, none with thrombus within. Thoracic tomography showed a millimetric nodule in the left lower lobe of the left lung. That finding was not considered metastatic, and a followup was deemed suitable for it. The patient was operated with left radical nephrectomy. Macroscopically, the nephrectomy material together with the surrounding fatty tissue weighed approximately 1500 grams. It was noted that the intact kidney tissue was restricted to a small area, and other areas were completely involved by the tumoral formation. The tumor was measuring 14 × 12 × 11 cm. It had a central necrotic zone on cross-section and was discharging a serohemorrhagic fluid. The tumor was observed to be well demarcated from the intact renal tissue by a smooth border and to have a pseudocapsule. Microscopically, tumoral cells had ovoid, round nuclei, coarse chromatin structure, and a narrow eosinophilic cytoplasm (Figure 2). The tumor contained patchy areas of necrosis. Two to three mitoses were counted on 10 high power fields. In immunohistochemical studies, tumoral cells showed strong reaction with CD34 and CD99. Less than 1% of cells showed nuclear positivity with Ki-67 (Figure 3). Despite repeated stainings with vimentin, no clear tumor evaluation could be made due to artifacts. The tumor was negative with Bcl-2, desmin, HMB-45, S100, FVIII, and CD31. Ewing sarcoma could not be ruled out. Molecular studies revealed no fusion related to the diagnosis of Ewing sarcoma. Histopathological, immunohistochemical findings, and molecular studies made the diagnosis of a solitary fibrous tumor. The patient is now currently free of disease at the 26th month of followup. 

## 3. Discussion

Solitary fibrous tumor is a rare mesenchymal tumor. It is mostly of pleural origin [4]. Nonetheless, extrapleural cases have also been reported. So far, the tumor has been reported to originate from upper respiratory system, liver, nasal cavity, paranasal sinuses, orbita, mediastinum, greater salivary glands, mammary glands, lung, meninges, and urogenital organs [5]. A few of the reported cases in the literature originated from the kidneys. Among the tumors of renal origin, 15% were located in renal capsule, 6% in peripelvis, and 3% in renal pelvis, while 76% had an unknown site of origin [6]. In our case, we also did not localize the exact site of origin. Nearly 50 cases of SFT of the kidney have been reported so far in the literature. Except for one case of a male patient of pediatric age group, all cases were adult patients [7]. Roughly 10%–15% of all SFTs are malignant [8]. The criteria for malignancy include increased cellularity, pleomorphism, increased mitotic activity (more than 4 mitoses on 10 high power fields), necrosis, and hemorrhage [8]. Only 3 of the tumors reported in the literature had necrosis and hemorrhage [9]. In our case, 2-3 mitoses on 10 high power fields may be interpreted to indicate its benign character. However, necrosis and hemorrhage therein require being cautious about its malignant potential. Nevertheless, histopathological prediction of an aggressive behavior of the tumor is difficult. The most common clinical symptom is a blunt pain on the side of the mass. Macroscopic hematuria may accompany that pain. Our case also presented with blunt flank pain. A palpable mass and microscopic hematuria were detected. Macroscopically, SFTs are well-demarcated, solid lesions with a gray-whitish color on cross-section that do not usually contain necrosis or hemorrhage. Microscopically, they show long spindle cell proliferation. They contain patchy hypo- and hypercellular areas. Cytoplasms of spindle cells appear small and ill-bordered. They are highly vascular tumors [6]. Our case was characterized by increased vascularity. Electron microscopy shows cells with fibroblastic features. Electron microscopy is beneficial in differentiating the tumor from neoplasms with nervous and muscular components. Immunohistochemical studies show strong staining of the tumor cells with CD34, Bcl-2, CD99, and vimentin. They are nonreactive with keratin, actin, S100, desmin, CD117, epithelial membrane antigen, FVIII, and D2-40. With Ki-67, 2%-3% of cells show nuclear positivity. CD34 and CD99 positivities are present in 90%–95% and 70%–75% of cases, respectively [9]. Bcl-2 positivity is encountered in 20%–35% of cases. Vimentin positivity has a rate of 90%–95% [10]. We observed CD34 and CD99 positivities and a positive reaction with Ki-67 in less than 1% of cells in our case. 

SFTs must be differentiated from the malignant and benign spindle cell tumors of the kidney. It is particularly difficult to differentiate it especially from hemangiopericytoma (HPC) that has similar histological findings and CD34 positivity. Nearly 30 cases of renal HPC have been reported in the literature. HPCs macroscopically contain hemorrhagic areas. Compared to STFs, HPCs have less cellular diversity and stromal hyalinization. While CD34 positivity is weak in HPC, it is diffuse and strong in SFT [11]. Other tumors from which SFTs are hard to be distinguished are fibroma, angiomyolipoma, leiomyoma, schwannoma, neurofibroma, and sarcomatoid renal cell carcinoma. In conclusion, immunohistochemical examination is the main diagnostic method. SFTs generally have a favorable prognosis. A majority of them do not recur or metastasize [11]. Malignant behaviors such as metastasis and recurrence are observed in 10% of extrapleural tumors and 10%–15% of intrapleural tumors [12]. Malignant behavior has been reported in a limited number of cases in the literature [13–15]. A careful and adequate resection is the mainstay of therapy of renal SFT. The role of adjuvant chemotherapy is still unclear. Given that it is difficult to predict the aggressive clinical behavior of the tumor, it is of paramount importance to follow the patients at a regular basis. We did not identify any recurrence or metastasis at the 26th month of followup following surgery in our patient. 

## 4. Conclusion

Solitary fibrous tumor is a rare mesenchymal tumor with good prognosis. Its definitive diagnosis can be made with pathological studies. It may be confused with malignant tumors. The pathologists must be careful in this respect.
